# Alternative splicing of the Wnt trafficking protein, Wntless and its effects on protein-protein interactions

**DOI:** 10.1186/s12860-019-0208-1

**Published:** 2019-07-08

**Authors:** Jessica Petko, Mathura Thileepan, Molly Sargen, Victor Canfield, Robert Levenson

**Affiliations:** 1 Biology Department, Penn State York, York, Pa USA; 2 Department of Pharmacology, Penn State College of Medicine, Hershey, PA USA

**Keywords:** Wnt, Wntless, Protein-protein interaction, Opioid receptor, Adenosine receptor, Yeast 2-hybrid

## Abstract

**Background:**

Wntless (Wls) is a protein that regulates secretion of Wnt signaling molecules from Wnt-producing cells. Wnt signaling is known to be critical for several developmental and homeostatic processes. However, Wnt-independent functions of Wls are now being elucidated. Primates express an alternative splice variant of Wls (here termed WlsX). WlsX contains an alternatively spliced COOH-terminus, and does not appear to be able to sustain significant levels of WNT secretion because of its inability to undergo retrograde trafficking to the endoplasmic reticulum. The functional significance for this alternatively spliced form of Wls has not yet been elucidated. We previously identified a cohort of Wls interacting proteins using a combination of yeast 2-hybrid and candidate gene approaches.

**Results:**

In the present study, we analyzed the interaction of WlsX with previously identified Wls interactors, and additionally screened for novel protein interactors of WlsX utilizing a membrane yeast two hybrid screen. Three novel Wls interactors, Glycoprotein M6A (GPM6A), Alkylglycerol Monooxygenase (AGMO), and ORAI1 were identified. Each of these novel WlsX interactors, as well as all other Wls interacting proteins identified previously, with the exception of the mu-opioid receptor, were found to interact with both Wls and WlsX splice forms. We show that WlsX can form homodimers, but that WlsX may not interact with Wls.

**Conclusions:**

WlsX has the ability to form homodimers and to interact with most known Wls interacting proteins. Taken together, our results suggest that Wls and WlsX may have overlapping, but distinct functions, including sensitivity to opioid drugs. While studies have focused on the ability of Wls interacting proteins to affect Wnt secretion, future efforts will explore the reciprocal regulation of these proteins by Wls, possibly via Wnt-independent mechanisms.

## Background

Wnts serve as major signaling molecules for a number of developmental processes such as establishment of planar cell polarity, body axis, cell fate, and differentiation. In addition to its role in development, Wnt signaling remains an important regulator of adult tissue homeostasis, maintaining synapses in the normal adult brain, whereas dysfunctions in Wnt signaling lead to several types of cancers [1–10]. Regulation of Wnt signaling can occur at two major control points. The first control point is in Wnt biosynthesis and/or release of Wnt proteins from Wnt producing cells. The second point of control is the signaling pathways which are activated in the Wnt recipient cell. While Wnt-induced signal cascades (canonical beta-catenin pathway, planar cell polarity pathway, and the calcium pathway) are well established, only recently has the biology of Wnt secretion received much attention [10–12]. Wnts are synthesized and lipid modified in the endoplasmic reticulum before trafficking through the *trans*-Golgi network to the cell surface for secretion [13–15]. Once modified, an eight-pass transmembrane protein, Wntless (Wls) associates with Wnt molecules in order to ensure proper packaging into release vesicles [16–18].

Some studies have shown that Wnt containing vesicles can be further processed into exosomes [19–22]. This may aid in the dispersal of Wnt signals from their original source to more distant paracrine targets than can be achieved by simple diffusion through extra cellular matrix, as Wnt proteins alone are hydrophobic due to lipid processing. Interestingly, it appears that Wls can also be packaged into these exocytic vesicles and transported between cells. Korkut et al. showed that GFP labeled Wls molecules expressed in motor neurons could cross the neuromuscular junction and localize to muscle tissue in *Drosophila* [23].

As Golgi-derived vesicles fuse with the plasma membrane, thereby releasing Wnts through conventional exocytotic mechanisms, Wls itself remains embedded in the plasma membrane of the releasing cell. However, rather than being degraded through endocytic trafficking to lysosomes, a large number of studies have described a retrograde recycling mechanism by which Wls proteins can be recruited for multiple rounds of Wnt secretion [24–29]. Following clathrin-mediated endocytosis, Wls is retrieved from early endosomes by the retromer complex and sorting nexin-3 and escorted back to the *trans*-Golgi network [27]. Yu et al. have shown that Wls is subsequently transported back to the endoplasmic reticulum in an ER-Golgi intermediate compartment (ERGIC)-dependent manner [30]. This membrane to ER trafficking pathway is used by exogenous toxins and secreted molecules. To our knowledge, Wls is the first transmembrane protein to demonstrate PM to ER trafficking. Internalization and retrieval of Wls by retromer requires a portion of the third intracellular loop of Wls, while Golgi to ER transport of Wls requires a conserved set of amino acids at the C-terminus of the protein [30, 31].

Yu et al. also characterized an alternative splice variant of Wntless that appears to be specific to primate species. This splice variant utilizes an alternative terminal exon leading to an altered 3′ UTR and C-terminus compared to Wls. In this manuscript, we refer to this primate-specific splice variant of Wls as WlsX. WlsX is widely expressed at low levels in human tissues, while the more conserved Wls isotype is expressed at much higher levels [30]. In cultured cells, WlsX localizes to the plasma membrane and cytosolic vesicles and unlike Wls, its expression does not appear to support detectable levels of Wnt3A secretion. However, ER recycling and capacity for Wnt secretion is partially restored if the KEAQE amino acid motif from Wls is fused to the C-terminus of WlsX. The physiological relevance of this splice variant is currently unknown.

We previously demonstrated that Wls interacts with the mu opioid receptor (MOR), and that morphine can dampen Wnt secretion, presumably through an inhibition of Wls recycling [32–35]. Having developed an interactome map for Wls [36], we discovered that Wls interacts with a number of proteins related to reward including the MOR, the A2A adenosine receptor, the dopamine transporter, and cannabinoid receptors. It is not known whether any of these proteins maintain their interaction with WlsX or whether WlsX interacts with a distinct subset of proteins within its unique C-terminus.

In the current study, we utilized a membrane yeast 2-hybrid (MYTH) screen to characterize the WlsX interactome. Using this approach, we identified three novel Wls interacting proteins that appear to interact with both Wls and WlsX. Interestingly, the only unique Wls interacting protein we have identified is the mu-opioid receptor (MOR) which interacts with Wls and not WlsX. We have previously shown that the Wls/MOR interaction contributes to the regulation of Wnt secretion (Jin et al). Our results therefore suggest that WlsX most likely does not play a role in regulating Wnt secretion, but may be functionally distinct from Wls.

## Results

### WlsX expression across species

**Fig. 1:**
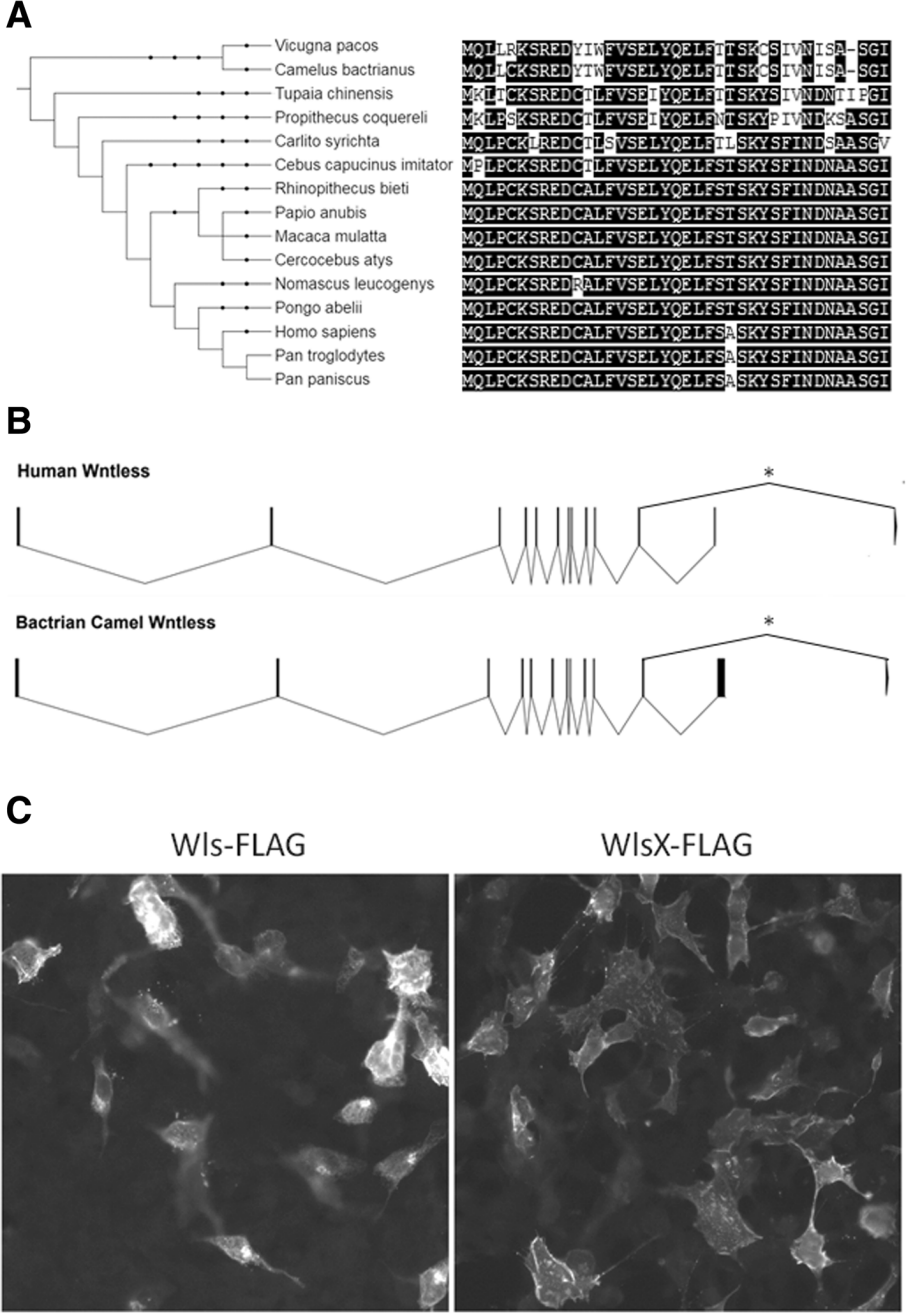
Phylogeny, gene structure, and subcellular localization of WlsX. **a** Phylogenetic tree with amino acid sequence comparison of the alternative C-terminal exon for all species of primate and camelid identified in an EST database search. Alignment corresponds to amino acid 504 to the C-terminal end of human WlsX (NP_079187.3). The tree was generated using the Interactive Tree of Life webpage (https://itol.embl.de/) and taxonomic information from the National Center for Biotechnology information (NCBI). Black dots represent internal nodes for which branching is collapsed. **b** Exon intron structure of Human and Bactrian Camel. The alternatively spliced 3′ exon is indicated with an asterisk (*). **c** Immunofluorescence image of HEK293T cells exogenously expressing FLAG-tagged Wls or WlsX

In a search of the National Center for Biotechnology Information (NCBI) Expressed Sequence Tag (EST) database, we identified transcripts for WlsX in a number of primate species as previously reported [30]. In addition, ESTs for WlsX were also identified in two members of the family Camelidae, alpacas and the Bactrian camel (Fig. 1a). The exon/inton structure is similar between humans and camels (Fig. 1b). We also confirm the previous observation by Yu et al. that WlsX maintains a peripheral pattern of expression consistent with plasma membrane localization, while Wls is more broadly distributed (Fig. 1c) when exogenously expressed in HEK 293 T cells.

### WlsX interacts with a set of previously identified Wls interactors

**Fig. 2:**
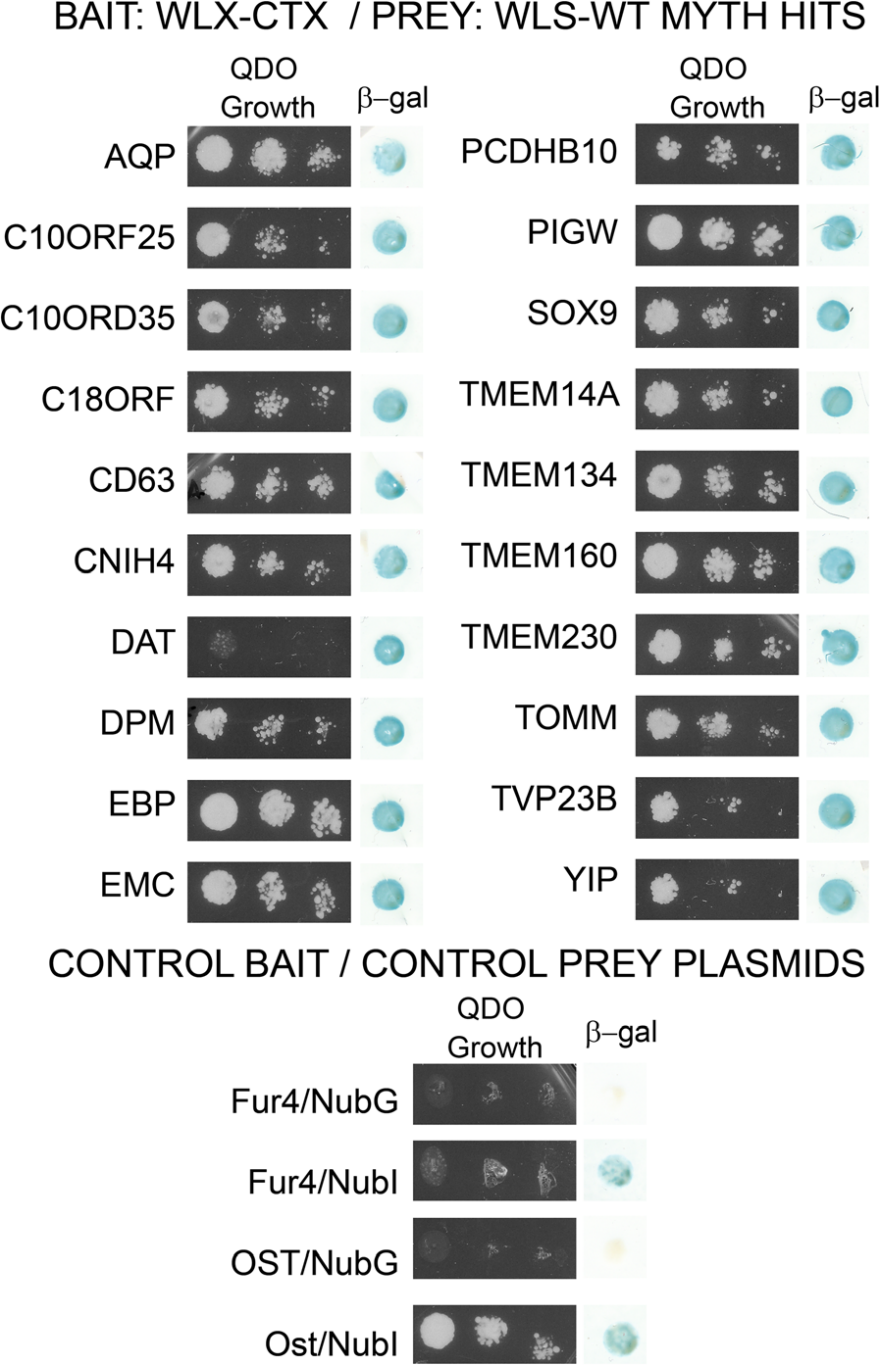
Directed MYTH screen testing the association of WlsX with known Wls interactors. Wls interactors identified in previous MYTH screens were assayed for interaction with WlsX. Growth on quadruple drop out media was tested in increasing dilutions (left panel for each). Beta-galactosidase activity was assessed by a filter lift assay (right panel for each). Positive controls linked to NubI show growth and beta-galactosidase activity while negative controls linked to NubG showed no growth or beta-galactosidase activity

We previously characterized members of the Wls interactome using membrane yeast 2-hybrid (MYTH) and traditional Y2H screening methods [36]. To determine whether alternative splicing within the C-terminal tail of Wls affects the association of Wls with these previously identified interactors, we performed MYTH assays utilizing full length WlsX as the bait protein. As shown in Fig. 2, WlsX does not appear to auto-activate the MYTH system as it does not interact with Fur4 or Ost fused to NubG. The same two proteins fused to NubI serve as a positive control, as this version of the ubiquitin N-terminus spontaneously associates with the C-terminus of ubiquitin fused to the bait protein.

We tested the interaction of WlsX with twenty prey constructs isolated in the previous Wls MYTH screen [36]. This includes several proteins that are involved in protein modification, intracellular trafficking, and developmentally-related processes (see Fig. 2). Although we did not know where in the aa sequence these proteins interacted with Wls, we hypothesized that if any of the proteins interacted with the C-terminal tail of Wls, they should not interact with WlsX due to the divergence in C-terminal amino acid sequence and structure between the two isoforms. As shown in Fig. 2, yeast that were co-transformed with WlsX bait and any of the prey constructs from the original Wls MYTH screen grew on quadruple drop out media and demonstrated beta-galactosidase activity. Growth on selective media was slightly lower for the dopamine transporter, however beta-galactosidase activity was just as robust as colonies containing the other prey plasmids. In our previous study, similar growth and lift patterns were described for DAT and Wls, and the interaction was confirmed using GST-pulldown and co-immunoprecipitation methods. It should be noted that growth on selective media and activity of beta-galactosidase are not always correlated. Nutritional selection on quadruple drop out media requires the activation of four genes. However, yeast grown for beta-galactosidase lift assays do not require interaction for growth and colonies are grown to high densities before testing. If the interaction is weaker, one might expect reduced levels of beta-galactosidase activity (lighter blue), however, this is not always the case and colonies may develop the same level of color with extended incubation. Our results indicate that proteins identified in the original MYTH screen also interact with WlsX, suggesting that the sites of interaction for these proteins most likely does not occur in the C-terminal domain of Wls, but more likely occur within an internal loop or transmembrane domain of the Wls isoforms.

### Screen for WlsX specific interactors

**Fig. 3:**
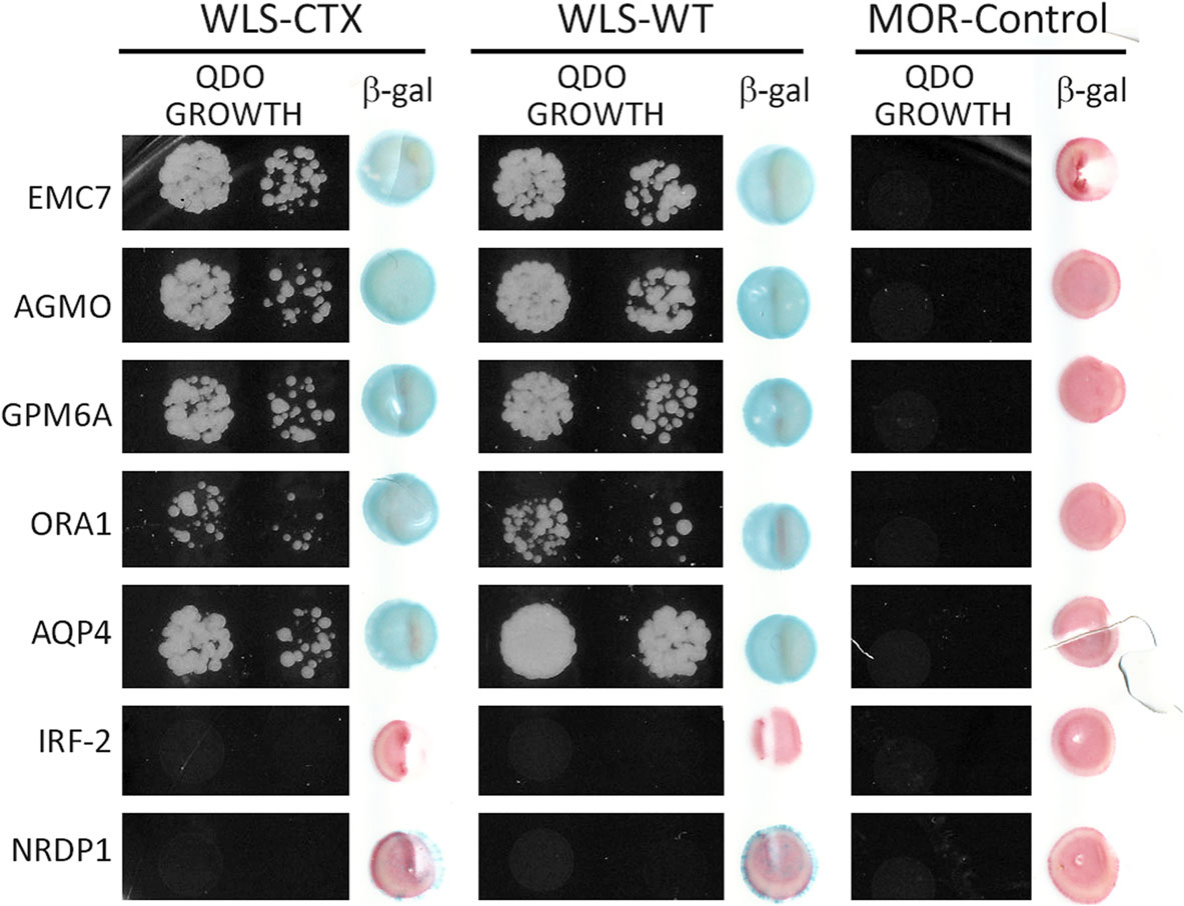
Directed MYTH assay testing the association of WlsX potential interactors from the WlsX MYTH screen. WlsX interactors identified in the current MYTH screen were tested for interaction with WlsX (Wls-CTX), Wls (Wls-WT), and the Mu Opioid Receptor (MOR – negative control). Growth on quadruple drop out media was tested in increasing dilutions (left panel for each). Beta-galactosidase activity was tested using a filter lift assay (right panel for each)

To identify novel WlsX interactors, we first performed a traditional yeast 2-hybrid screen utilizing only the sequence unique to the WlsX splice variant as bait. However, we discovered that the C-terminal tail of WlsX auto-activated the traditional Y2H assay by constitutively inducing reporter gene expression in the absence of prey proteins (data not shown). Since we previously have shown that the full length WlsX does not auto-activate the MYTH system, we performed a MYTH screen using the full-length WlsX isoform as bait to screen a human fetal brain cDNA library for unique WlsX interacting proteins [36]. This screen resulted in the identification of seven potential interactors, two of which (AQP4 and EMC7) were identified as Wls interactors in our previous MYTH screen [36]. Three novel protein interactors, AGMO, GPM6A, and ORAI1a, were confirmed to interact with WlsX in the MYTH system through independent retransformation of individual bait and prey proteins, followed by a positive beta-galactosidase reaction, and growth on quadruple drop-out nutritional selection (Fig. 3). In addition, these three proteins also interacted with full-length Wls. Two clones from the initial WlsX screen, IRF-2 and NRDP1, failed to interact with WlsX, Wls, or MOR upon retransformation. The red coloring of the colonies that are negative for beta-galactosidase activity is due to the buildup of an intermediate product in the adenine synthesis pathway. These results indicate that proteins identified in the WlsX MYTH screen are not specific to WlsX but also interact with Wls, suggesting that the sites of interaction for these new interactors most likely reside within domains that are conserved between Wls and WlsX. Because these proteins do not interact with the MOR, it appears that none of the five prey proteins we identified in the WlsX screen are capable of auto-activating the MYTH system.

**Fig. 4:**
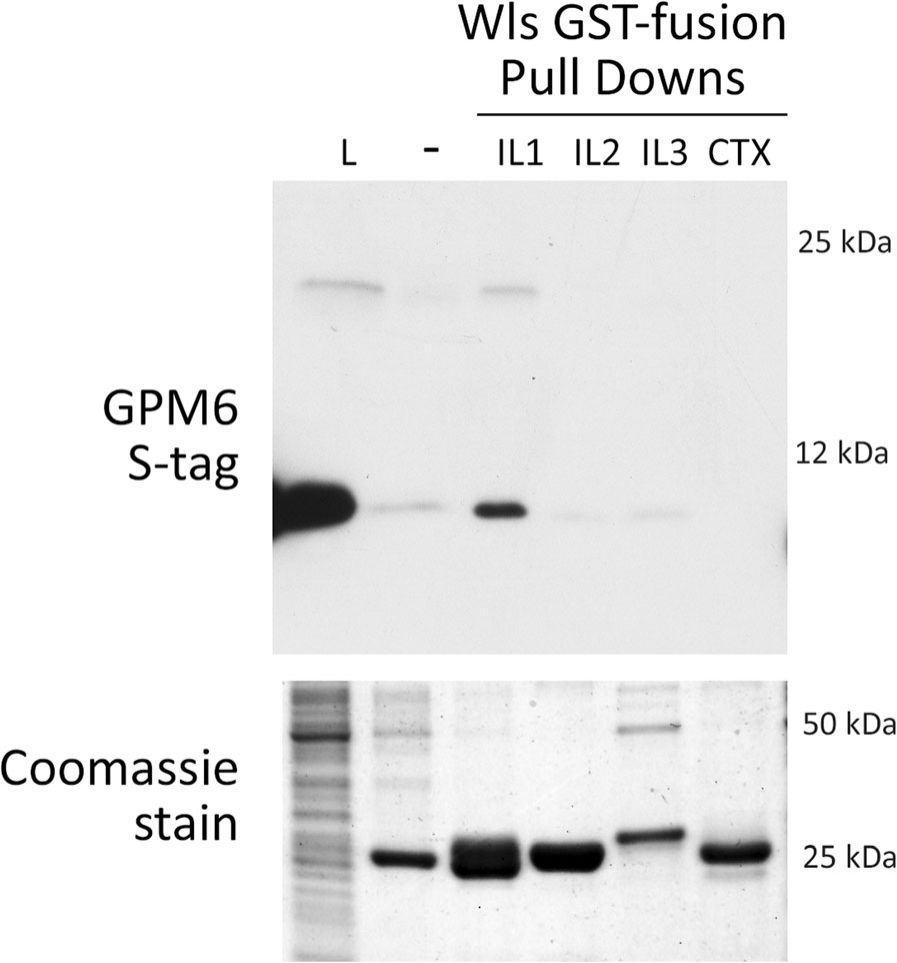
GST-pulldown and mapping of WlsX/GPM6 interaction. Western blot (top panel) was probed with an HRP-conjugated S-tag antibody. A small sample from each of the GST pulldowns was run on a separate gel for coomassie stain and pulldown verification. The second lane represents a control pulldown in which the beads were coated in GST only

Of the three newly confirmed Wls and WlsX interactors, we were especially interested in GPM6a. GPM6a is a membrane glycoprotein that promotes mu-opioid receptor endocytosis and facilitates receptor sorting into the recycling pathway [37]. To confirm and map the interaction sites between Wls and GPM6a we performed GST pulldowns using the intracellular portions of Wls as bait to precipitate the intracellular C-terminus of GPM6a (the intracellular portion of the protein found in the MYTH prey construct). As shown in Fig. 4, GPM6a appears to specifically co-precipitate with the first intracellular loop of Wls. On the western blot S-tagged GPM6a runs just under 12 kDa as expected, but there also appears to be a higher molecular weight band in the lanes containing this protein. Although these are denaturing gels, it is possible that the C-tail of GPM6a homodimerizes to give rise to a second band at a molecular weight of twice the monomer. These pulldown results confirm the interaction of Wls and GPM6a and suggest that the interaction lies within the first intracellular loop and the C-terminus, respectively.

### Interaction of WlsX with known Wls C-tail interactors

**Fig. 5:**
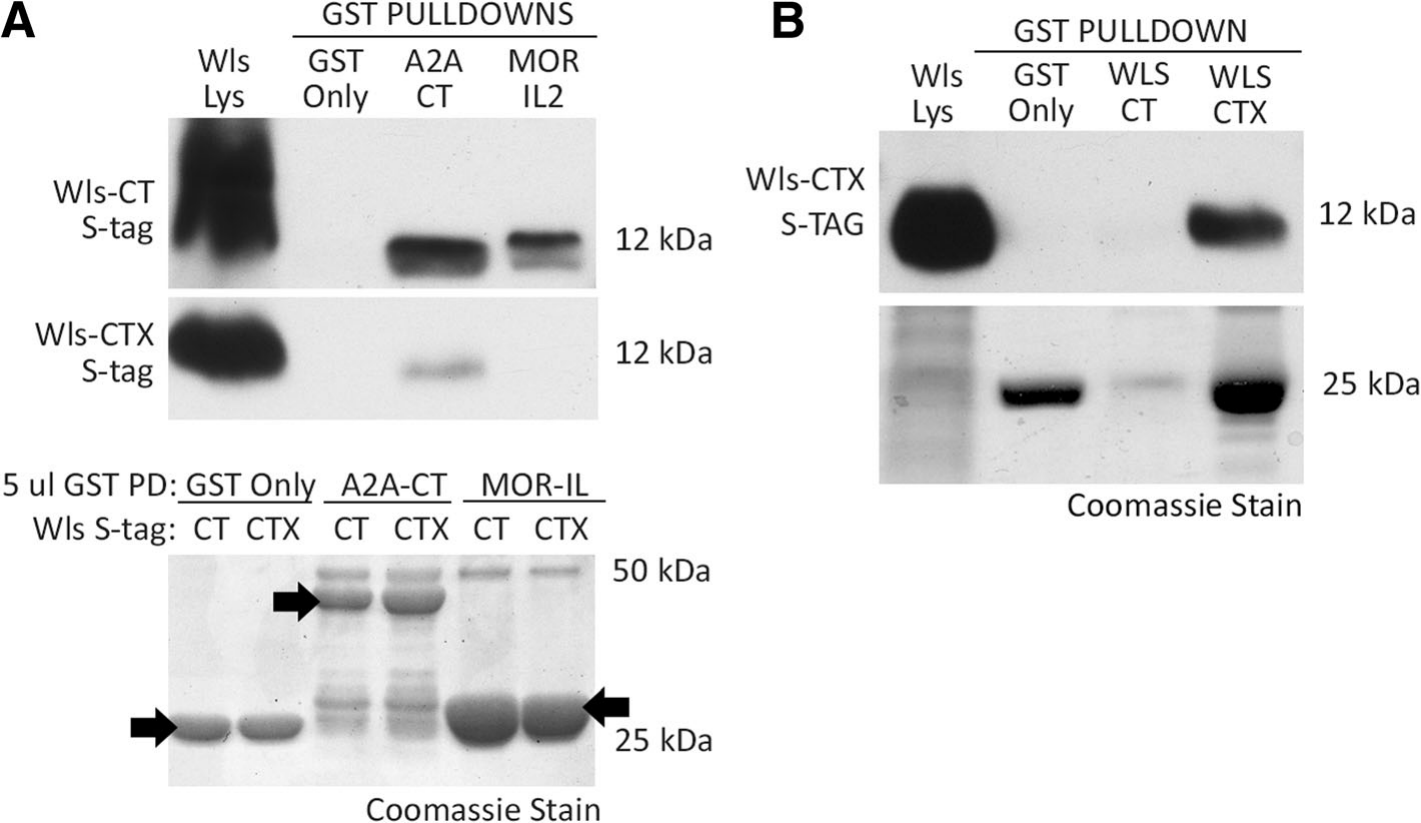
GST-pulldown analysis of WlsX homodimerization and heterodimerization with A2a Adenosine receptor (A2A), Mu opioid receptor (MOR), and Wls. **a** GST pulldown of GST-tagged A2A (C-tail) or MOR (intracellular loop 2-IL2) with S-tagged Wls splice variants. **b** GST pulldown analysis of GST-tagged Wls or WlsX with S-tagged WlsX. Blots for the top two panels in A and the top panel in B are probed with HRP conjugated S-tag antibody. A small sample from each of the GST pulldowns was run on a separate gel for coomassie stain and pulldown verification (bottom panels for **a** and **b**)

Wls was identified as an interactor of the mu opioid receptor (MOR) in a MYTH screen using MOR as bait [32, 34]. In a previous study, we tested another G-protein coupled receptor, the A2A-adenosine receptor (ADORA2A) for its interaction with Wls using GST pulldown and co-immunoprecipitation approaches [34]. For the MOR, the MOR/Wls interaction domains localized between the second intracellular loop of the MOR and the C-terminal tail of Wls [32, 34]. In contrast, ADORA2A and Wls appear to interact with one another through their respective C-terminal tail domains [36]. Additionally, we demonstrated that Wls can homodimerize through its C-terminal domain. Because these interactions all involve the C-terminal tail of Wls, we hypothesized that WlsX should not be able to heterodimerize with ADORA2A and MOR, or homodimerize with Wls. To test this hypothesis, the C-terminus of ADORA2A (A2A-CT) and the second intracellular loop of MOR (MORIL2) were fused to GST and tested using the GST-pulldown assay for their ability to precipitate the S-tagged C-terminal tail of Wls or WlsX. As shown in Fig. 5a, alternative splicing within the C-terminal tail prevents interaction of WlsX with the MORIL2. Conversely, the A2A adenosine receptor was able to pull down the C-tail of both Wls and WlsX. Therefore, it is likely that Wls interacts with the MOR through a portion of the C-tail that is alternatively spliced, while A2A interacts with a more proximal portion of the Wls C-tail that is conserved between Wls and WlsX.

Using a GST-pulldown assay, we also tested the ability of the WlsX C-tail to homodimerize with itself or to dimerize with C-tail of Wls. As shown in Fig. 5b, the C-tail of Wls was unable to pull down the C-tail of WlsX. However, the C-tail of WlsX was able to pull down an S-tagged version of the same protein domain. As seen in the Coomassie stain in Fig. 5b, the C-tail of Wls was pulled down to a much lesser extent than the C-tail of WlsX, which may partially explain the lack of evidence for a heterodimer of the splice variant products. These results suggest that like Wls, WlsX can homodimerize through its C-terminal tail, but that the two isoforms (Wls and WlsX) may not dimerize with one another.

## Discussion

In this report, we analyzed the WlsX interactome. WlsX is an alternative splice variant of Wls that differs from Wls in its C-terminal amino acid sequence. ESTs for the alternative splice variant of WlsX were identified in multiple primate species as well as two members of the family Camelidae (camels). However, we failed to identify WlsX in rodents, or in any other animal species ancestral to primates, suggesting that WlsX must have arisen fairly late in mammalian evolution. As previously described by Yu et al. [30], we find that compared to Wls, which is localized primarily in the ER and Golgi, WlsX is localized predominantly at the plasma membrane. MYTH screening and GST-pulldown analysis were used in an initial approach to characterize the WlsX interactome. Three novel WlsX interactors were identified in the MYTH screen including Glycoprotein M6A (GPM6A; a membrane protein involved in axon outgrowth and neuronal signaling regulation), Alkylglycerol Monooxygenase (AGMO; a protein involved in synthesis of specific membrane lipids), and ORAI1 (an ER calcium channel involved in the sequestration of calcium from the cytoplasm to the ER lumen). However, these proteins were also found to interact with Wls. In fact, of all the previously identified Wls interacting proteins, only the mu opioid receptor failed to interact with WlsX. This result suggests that Wls and WlsX may have overlapping, but distinct functions, including sensitivity to opioid drugs.

## Conclusions

WlsX does not support significant levels of Wnt secretion, which leads to the question of its cellular function [30]. Recently a cell autonomous and Wnt-independent role for Wls in dendrite self-avoidance has been reported in *C. elegans* and Drosophila [38]. Dendritic self-avoidance was localized to the second extracellular loop of Wls, whereas the first extracellular loop of Wls appears to be required for regulating Wnt secretion. While dendritic self-avoidance is independent of Wls’s ability to regulate Wnt secretion, it does require Wiskott-Aldrich syndrome protein (WASP)-dependent actin assembly. These findings raise the question of whether there are other Wnt-independent roles for Wls. Although the vast majority of protein interactors are shared between Wls and WlsX, the fact that WlsX doesn’t interact with the mu-opioid receptor, nor does it support Wnt secretion, provides evidence for the possibility that WlsX does in fact possess novel functional properties. One possibility is that WlsX acts as a dominant negative or regulatory form of Wls. While it can bind most Wls interactors, it does not normally recycle for continued Wnt release. Does the internalization of WlsX interactors drive WlsX internalization? Conversely, no studies have looked at the effects of Wls on recycling dynamics of GPCRs. This information may be a key to understanding whether WlsX plays some sort of dominant negative role in trafficking processes since it appears to not undergo detectable internalization and recycling. This would not be the case with MOR, since WlsX does not associate with this protein, but may be the case for other GPCRs such as the adenosine receptor.

One of the newly identified WlsX interacting proteins is GPM6a, a tetraspanin domain-containing protein that is highly expressed in developing neurons, particularly throughout axonal growth cones [39–52]. While this interaction was not specific to WlsX, as it also interacted with Wls, its known functions make it an interesting candidate for future study of Wls and WlsX function. GPM6a is thought to aid in the formation of filopodium through a mechanism involving lipid raft localization and the Rac1/Pak1 signaling pathway [51]. Overexpression of GPM6a results in an increase in neurite formation while knockdown reduces neurite outgrowth [52]. Intracellular regulation of filipodium dynamics requires the C-terminal intracellular domain of GPM6a, the portion of this protein shown in this study to associate with Wls and WlsX.

GPM6a has also been shown to interact with the MOR and regulate its internalization [37, 53]. In cultured cells, overexpression of GPM6a facilitated both constitutive receptor internalization and agonist-dependent internalization, while overexpression of a truncated GPM6a protein blocked both forms of MOR internalization. Interaction sites were mapped to a portion of GPM6a spanning transmembrane domains 3 and 4 (including the second extracellular loop) and an area of the mu opioid receptor spanning transmembrane domains 4–6 (including extracellular loop 3 and intracellular loop 3). GPM6a was also observed to interact with the delta opioid receptor, the cannabinoid type 1 receptor, and the somatostatin receptor ss2A, but not metabotropic glutamate receptors [53]. Similarly, we have previously shown that Wls interacts with the MOR, DOR, and cannabinoid receptors type 1 and 2 albeit through different regions of the proteins (C-tail of Wls with IL2 of opioid receptors) [34, 36]. Opioid drug-induced internalization of MOR also drives the internalization of Wls in cells and in rat striatal dendrites [32, 54]. Morphine, an opioid agonist that delays MOR internalization, increases cell surface localization of Wls and decreases Wls recycling, resulting in an overall decrease in Wnt secretion [32–34, 54]. Future studies will assess whether GPM6a alters the constitutive recycling of Wls and whether GPM6a is important for co-internalization of Wls and MOR in cells treated with opioids that promote robust internalization. Additionally, one might ask whether GPM6a could increase the internalization of MOR in the presence of morphine, as an opioid that delays internalization.

While many studies have investigated the Wnt-dependent role of Wls in various biological processes, few have explored whether Wls has other Wnt-independent roles. For example, it is unknown whether Wls can affect the function of the proteins it interacts with at the plasma membrane, such as GPCRs and the dopamine transporter. This information may help to elucidate whether WlsX is produced as a regulatory form of Wls or whether it has its own novel function unrelated to those of Wls.

## Methods

### Cell culture and immunofluorescence

Human embryonic kidney 293 T (HEK-293 T; obtained from ATCC - catalog number CRL-3216) cells were cultured in Dulbecco’s Modified Eagle Medium (DMEM) supplemented with 1% PenStrep and 10% fetal bovine serum. Wls and WlsX were subcloned into the pCMV-Tag 2 (FLAG) expression vector and transfected into the HEK-293 T cells using Effectene transfection reagent (Qiagen, Valencia, CA).For immunofluorescence, cells were grown on poly-lysine coated coverslips, washed with Dulbecco’s phosphate buffered saline (DPBS), fixed with 4% paraformaldehyde, permeabilized with 0.2% Triton X-100 in PBS containing 10% normal donkey serum (Jackson ImmunoResearch), and stained with the a rabbit anti-FLAG antibody. The coverslips were mounted onto glass slides using Prolong Gold Anti-Fade Mountant (Life Technologies). Fluorescent images were captured with an inverted fluorescent microscope (Nikon Eclipse TE2000-S).

### Membrane yeast 2-hybrid (MYTH)

#### Bait strain construction

Membrane yeast 2-hybrid (MYTH) techniques are utilized to identify novel interactions between membrane-localizing proteins [55, 56]. In this assay, membrane proteins are fused to the C-terminal or N-terminal half of ubiquitin are used to create bait and prey constructs, respectively. Additionally, the bait protein is fused to a  transcription factor such as LexA. Upon interaction, the ubiquitin molecule is reconstituted, and a ubiquitin-specific protease cleaves and releases the transcription factor from the bait protein. The transcription factor can then enter the nucleus to activate transcription of reporter genes. In this study, reporters include genes required for the synthesis of essential amino acids and nucleotides (Leu,Trp,His,Ura) and LacZ which encodes beta-galactosidase. For this study, the open reading frames of the bait proteins (Wls, WlsX, or the mu opioid receptor) were subcloned into the bait plasmid pCCW-STE (Dualsystems Biotech AG, Switzerland) and individually transformed into the THY.AP4 yeast strain to create bait strains. Wls and MOR have previously tested negative for bait auto-activation. The WlsX bait strain was tested for auto-activation by co-transforming it with empty prey vector, positive control plasmids (containing Fur-NubI or Ost-NubI), or negative control plasmids (containing Fur-NubG or Ost-NubG). Control plasmids were a gift from Igor Stagljar.

#### MYTH screening

For the MYTH screen, the WlsX bait strain was co-transformed with a human fetal brain cDNA library (Dualsystems) and grown on quadruple dropout selection (−Leu,-Trp,-His,-Ura) plates containing 3-AT. Resulting colonies were subjected to nitrocellulose lift assays for interaction confirmation. 2.5 million clones were screened and 52 transformants grew under quad selection. All colonies tested positive for β-gal activity. Prey plasmids were isolated, sequenced, and identified via BLAST search of the NCBI nucleotide database.

#### Directed MYTH

cDNA clones encoding potential Wls interactors were retransformed into bait strain containing Wls, WlsX, or MOR to test the specificity of the interaction with WlsX and to assess whether the prey plasmids auto-activate reporter genes. Previously identified prey proteins from a MYTH screen performed with Wls were also tested for interaction with WlsX. Original prey clones from the Wls screen were transformed into the WlsX bait strain. For all directed MYTH assays, interaction was measured by assessing growth on quad drop out plates and β-gal activity in a nitrocellulose lift assay.

### GST pulldowns

The C-terminal portion of WlsX (Wls-CTX) was subcloned into the pGEX-4 T-1 (Amersham Biosciences, Piscataway, NJ) and the pET30 (Novagen, Madison, WI) bacterial expression plasmids to create GST- tagged or S-tagged proteins, respectively. GST-tagged Wls-IL1 (residues 254–268), Wls-IL2 (residues 325–331), Wls-IL3 (residues 402–431), Wls-CT (residues 493–541), and A2A-CT (residues 291–412) were prepared previously [36]. Proteins were synthesized via auto-induction as described by Studier et al. [57]. Briefly, pGEX-4 T-1 and pET30 plasmids were transformed into BL21 (DE3) *E.coli* cells and induced using ZYP-5052 auto-induction media. Bacterial proteins were extracted through sonication in PBS containing 1% Triton × 100 and protease inhibitors (cOmplete MINI EDTA free: Roche). Resulting lysates were cleared by centrifugation and stored at − 80 deg. C until use.

GST-tagged proteins were bound to glutathione sepharose beads (GE Healthcare, Piscataway, NJ) and then incubated with the indicated S-tagged proteins. Beads treated with GST alone served as a negative control. Proteins were eluted from the beads with loading dye containing SDS and β-mercaptoethanol, separated by SDS-page, and analyzed by western blotting with HRP-conjugated S-tag antibody (1:5000, Novagen, Madison, WI).

## Data Availability

The datasets used and/or analyzed during the current study are available from the corresponding author on reasonable request.

## Abbreviations

A2A-CT: C-terminus of ADORA2A
ADORA2A: A2A-adenosine receptor
AGMO: Alkylglycerol Monooxygenase
DMEM: Dulbecco’s Modified Eagle Medium
ERGIC: ER-Golgi intermediate compartment
EST: Expressed Sequence Tag
GFP: Green fluorescent protein
GPM6A: Glycoprotein M6A
GST: Glutathione S-transferase
HEK-293 T: Human embryonic kidney 293 T
HRP: Horseradish peroxidase
MOR: Mu-opioid receptor
MORIL2: Second intracellular loop of MOR
MYTH: Membrane yeast-2hybrid
SDS: Sodium dodecyl sulfate
Wls: Wntless

## References

[1] Dickins EM, Salinas PC (2013). Wnts in action: from synapse formation to synaptic maintenance. Front Cell Neurosci.

[2] Hussaini SM (2014). Wnt signaling in neuropsychiatric disorders: ties with adult hippocampal neurogenesis and behavior. Neurosci Biobehav Rev.

[3] Mulligan KA, Cheyette BN (2017). Neurodevelopmental perspectives on Wnt signaling in psychiatry. Mol Neuropsychiatry.

[4] Rosso SB, Inestrosa NC. WNT signaling in neuronal maturation and synaptogenesis. Front Cell Neurosci. 2013;(7):103. 10.3389/fncel.2013.00103 PMC3701138 23847469

[5] Laezza C (2013). Anandamide inhibits the Wnt/beta-catenin signalling pathway in human breast cancer MDA MB 231 cells. Eur J Cancer.

[6] Kim YM, Kahn M (2014). The role of the Wnt signaling pathway in cancer stem cells: prospects for drug development. Res Rep Biochem.

[7] Song L (2015). Development of small molecules targeting the Wnt signaling pathway in Cancer stem cells for the treatment of colorectal Cancer. Clin Colorectal Cancer.

[8] Sumithra B, Saxena U, Das AB (2016). Alternative splicing within the Wnt signaling pathway: role in cancer development. Cell Oncol (Dordr).

[9] Taciak B, et al. Wnt signaling pathway in development and cancer. J Physiol Pharmacol. 2018;69(2). 10.26402/jpp.2018.2.07 29980141

[10] Steinhart Zachary, Angers Stephane (2018). Wnt signaling in development and tissue homeostasis. Development.

[11] Langton PF, Kakugawa S, Vincent JP (2016). Making, exporting, and modulating Wnts. Trends Cell Biol.

[12] Takada S (2017). Differences in the secretion and transport of Wnt proteins. J Biochem.

[13] Herr P, Basler K (2012). Porcupine-mediated lipidation is required for Wnt recognition by Wls. Dev Biol.

[14] Willert K (2003). Wnt proteins are lipid-modified and can act as stem cell growth factors. Nature.

[15] Zhai L, Chaturvedi D, Cumberledge S (2004). Drosophila wnt-1 undergoes a hydrophobic modification and is targeted to lipid rafts, a process that requires porcupine. J Biol Chem.

[16] Banziger C (2006). Wntless, a conserved membrane protein dedicated to the secretion of Wnt proteins from signaling cells. Cell.

[17] Bartscherer K (2006). Secretion of Wnt ligands requires Evi, a conserved transmembrane protein. Cell.

[18] Goodman RM (2006). Sprinter: a novel transmembrane protein required for Wg secretion and signaling. Development.

[19] Chen Q (2016). Different populations of Wnt-containing vesicles are individually released from polarized epithelial cells. Sci Rep.

[20] Harada T (2017). Wnt5b-associated exosomes promote cancer cell migration and proliferation. Cancer Sci.

[21] Tassew NG (2017). Exosomes mediate mobilization of autocrine Wnt10b to promote axonal regeneration in the injured CNS. Cell Rep.

[22] Gross JC (2012). Active Wnt proteins are secreted on exosomes. Nat Cell Biol.

[23] Korkut C (2009). Trans-synaptic transmission of vesicular Wnt signals through Evi/Wntless. Cell.

[24] Belenkaya TY (2008). The retromer complex influences Wnt secretion by recycling wntless from endosomes to the trans-Golgi network. Dev Cell.

[25] Eaton S (2008). Retromer retrieves wntless. Dev Cell.

[26] Franch-Marro X (2008). Wingless secretion requires endosome-to-Golgi retrieval of Wntless/Evi/sprinter by the retromer complex. Nat Cell Biol.

[27] Harterink M (2011). A SNX3-dependent retromer pathway mediates retrograde transport of the Wnt sorting receptor Wntless and is required for Wnt secretion. Nat Cell Biol.

[28] Port F (2008). Wingless secretion promotes and requires retromer-dependent cycling of Wntless. Nat Cell Biol.

[29] Yang PT (2008). Wnt signaling requires retromer-dependent recycling of MIG-14/Wntless in Wnt-producing cells. Dev Cell.

[30] Yu J (2014). WLS retrograde transport to the endoplasmic reticulum during Wnt secretion. Dev Cell.

[31] Gasnereau I (2011). Identification of an endocytosis motif in an intracellular loop of Wntless protein, essential for its recycling and the control of Wnt protein signaling. J Biol Chem.

[32] Jin J (2010). Interaction of the mu-opioid receptor with GPR177 (Wntless) inhibits Wnt secretion: potential implications for opioid dependence. BMC Neurosci.

[33] Jin J (2010). Expression of GPR177 (Wntless/Evi/sprinter), a highly conserved Wnt-transport protein, in rat tissues, zebrafish embryos, and cultured human cells. Dev Dyn.

[34] Petko J (2013). MOR is not enough: identification of novel mu-opioid receptor interacting proteins using traditional and modified membrane yeast two-hybrid screens. PLoS One.

[35] Reyes BA (2012). Opiate agonist-induced re-distribution of Wntless, a mu-opioid receptor interacting protein, in rat striatal neurons. Exp Neurol.

[36] Petko J (2018). Identifying novel members of the Wntless interactome through genetic and candidate gene approaches. Brain Res Bull.

[37] Liang YJ (2008). Membrane glycoprotein M6A promotes mu-opioid receptor endocytosis and facilitates receptor sorting into the recycling pathway. Cell Res.

[38] Liao CP (2018). Cell-autonomous regulation of dendrite self-avoidance by the Wnt secretory factor MIG-14/Wntless. Neuron.

[39] Scorticati C, Formoso K, Frasch AC (2011). Neuronal glycoprotein M6a induces filopodia formation via association with cholesterol-rich lipid rafts. J Neurochem.

[40] Sato Y (2011). Induction of axon growth arrest without growth cone collapse through the N-terminal region of four-transmembrane glycoprotein M6a. Dev Neurobiol.

[41] Monteleone MC (2017). Neural glycoprotein M6a is released in extracellular vesicles and modulated by chronic stressors in blood. Sci Rep.

[42] Ito Y, Honda A, Igarashi M (2018). Glycoprotein M6a as a signaling transducer in neuronal lipid rafts. Neurosci Res.

[43] Honda A (2017). Extracellular signals induce glycoprotein M6a clustering of lipid rafts and associated signaling molecules. J Neurosci.

[44] Garcia MD (2017). The membrane glycoprotein M6a endocytic/recycling pathway involves Clathrin-mediated endocytosis and affects neuronal synapses. Front Mol Neurosci.

[45] Fuchsova B (2009). Cysteine residues in the large extracellular loop (EC2) are essential for the function of the stress-regulated glycoprotein M6a. J Biol Chem.

[46] Formoso K (2016). Evidence for a role of glycoprotein M6a in dendritic spine formation and synaptogenesis. Mol Cell Neurosci.

[47] Formoso K (2015). Filopodia formation driven by membrane glycoprotein M6a depends on the interaction of its transmembrane domains. J Neurochem.

[48] Formoso K (2015). Tyrosine 251 at the C-terminus of neuronal glycoprotein M6a is critical for neurite outgrowth. J Neurosci Res.

[49] Cooper B, Werner HB, Flugge G (2008). Glycoprotein M6a is present in glutamatergic axons in adult rat forebrain and cerebellum. Brain Res.

[50] Brocco MA, Fernandez ME, Frasch AC (2010). Filopodial protrusions induced by glycoprotein M6a exhibit high motility and aids synapse formation. Eur J Neurosci.

[51] Alvarez Julia A, Frasch AC, Fuchsova B (2016). Neuronal filopodium formation induced by the membrane glycoprotein M6a (Gpm6a) is facilitated by coronin-1a, Rac1, and p21-activated kinase 1 (Pak1). J Neurochem.

[52] Alfonso J (2005). The stress-regulated protein M6a is a key modulator for neurite outgrowth and filopodium/spine formation. Proc Natl Acad Sci U S A.

[53] Wu DF (2007). Membrane glycoprotein M6a interacts with the micro-opioid receptor and facilitates receptor endocytosis and recycling. J Biol Chem.

[54] Jaremko KM (2014). Morphine-induced trafficking of a mu-opioid receptor interacting protein in rat locus coeruleus neurons. Prog Neuro-Psychopharmacol Biol Psychiatry.

[55] Kittanakom S (2009). Analysis of membrane protein complexes using the split-ubiquitin membrane yeast two-hybrid (MYTH) system. Methods Mol Biol.

[56] Snider J, et al. Split-ubiquitin based membrane yeast two-hybrid (MYTH) system: a powerful tool for identifying protein-protein interactions. J Vis Exp. 2010;(36). 10.3791/1698 PMC2818708 20125081

[57] Studier FW (2005). Protein production by auto-induction in high density shaking cultures. Protein Expr Purif.

